# Long-term prognosis and postoperative liver atrophy after gastrectomy in patients with gastric cancer and liver cirrhosis

**DOI:** 10.1007/s00595-026-03471-1

**Published:** 2026-08-25

**Authors:** Atsushi Yasuda, Masuhiro Terada, Naoko Konami, Atsushi Yamada, Masashi Koda, Tomohiro Nakanishi, Yoko Hiraki, Hiroaki Kato, Osamu Shiraishi, Masayuki Shinkai, Motohiro Imano, Yutaka Kimura, Takushi Yasuda

**Affiliations:** 1 Department of Surgery, Kindai University Faculty of Medicine (Osaka Medical Campus), 1-14-1 Mihara-dai, Minami-ku, Sakai-shi, 590-0197 Osaka Japan; 2 Outpatient Chemotherapy Center, Kindai University Hospital, 1-14-1 Mihara-dai, Minami-ku, Sakai-shi, 590-0197 Osaka Japan; 3 Department of Surgery, Kindai-Nara Hospital, 1248-1 Otodacho, Ikoma, Nara, 630-0293 Japan

**Keywords:** gastric cancer, gastrectomy, liver cirrhosis, atrophy

## Abstract

**Purpose:**

Gastrectomy in patients with liver cirrhosis is associated with an increased postoperative risk and poor prognosis; however, the long-term outcomes remain unclear. This study evaluated the survival of patients with cirrhosis after gastrectomy, focusing on postoperative nutritional deterioration and time-dependent survival effects.

**Methods:**

This retrospective study included 847 patients who underwent gastrectomy for gastric cancer. Patients were categorized into liver cirrhosis (LC) and non-LC groups. One-to-four propensity score matching was performed to adjust for the baseline characteristics. Overall survival, cancer-specific survival, and postoperative liver volume changes were compared between the two groups. The time-dependent effects were examined using a piecewise-constant hazard model.

**Results:**

After matching, overall survival was significantly worse in the LC group, whereas the cancer-specific survival did not differ between the groups. The survival disadvantage was most pronounced within the first postoperative year. An exploratory analysis demonstrated significant postoperative hepatic atrophy in patients with cirrhosis. Liver disease-related mortality, including hepatic failure and hepatocellular carcinoma, was significantly higher in the LC group.

**Conclusions:**

Gastrectomy in patients with liver cirrhosis was associated with a worse overall survival, primarily because of liver disease-related mortality, whereas cancer-specific survival was comparable to that in patients without cirrhosis. A greater postoperative liver volume reduction has been observed in the patients with cirrhosis, although its mechanism and clinical significance remain unclear. Further prospective studies are warranted to determine whether nutritional or other perioperative interventions improve the postoperative outcomes.

## Introduction

Because the liver plays a crucial role in maintaining systemic homeostasis, surgical treatment in patients with coexisting liver disease is associated with a high risk of severe postoperative complications, including postoperative bleeding, liver failure, and hepatorenal syndrome, ultimately resulting in increased mortality rates [1–4].

In particular, gastrectomy with lymph node dissection requires direct ligation and division of portal collateral vessels, such as the short and left gastric veins, thereby imposing substantial surgical stress on patients with liver cirrhosis. Consequently, these patients are more susceptible to excessive intraoperative bleeding, postoperative ascites, lymphatic leakage, and liver function deterioration [5–7]. Gastrectomy in patients with cirrhosis has been suggested to carry higher rates of postoperative complications and mortality than other abdominal surgical procedures [1–4].

In recent years, advances in perioperative management and the development and refinement of surgical instruments have improved the safety of gastric cancer surgery in patients with liver cirrhosis [8–10]. However, these reports have not established whether such improvements in perioperative safety translate to a survival benefit. Accordingly, it remains unclear whether surgical intervention itself confers a clinically meaningful long-term prognostic advantage in patients with gastric cancer and concomitant liver cirrhosis, and the optimal treatment strategy for this high-risk population continues to be debated.

To address this issue, we conducted a retrospective study of patients who underwent gastrectomy for gastric cancer since 2002. Using 1:4 propensity score matching, we evaluated the long-term prognosis according to the presence or absence of liver cirrhosis. Furthermore, the prognosis of patients with liver cirrhosis was analyzed according to disease severity based on the Child–Pugh classification. This study aimed to clarify the prognostic impact of liver cirrhosis and its severity, and to identify potential contributory factors that may inform postoperative management strategies.

### Patients and methods

This retrospective study included 847 patients with gastric cancer who underwent gastrectomy at Kindai University Hospital between January 2000 and December 2024. Patients were divided into two groups according to the presence or absence of liver cirrhosis: the liver cirrhosis group (LC group; *n* = 25) and non-cirrhosis group (non-LC group; *n* = 822). Liver cirrhosis was defined based on a previous clinical diagnosis by hepatologists or the presence of findings suggestive of cirrhosis on preoperative laboratory and imaging evaluation. In all patients, intraoperative macroscopic findings consistent with cirrhosis, including a nodular liver surface, irregular hepatic contour, and blunt liver edge, were confirmed, and the diagnosis was made based on the overall clinical and intraoperative assessment. Patients with chronic hepatitis or hepatic fibrosis without sufficient clinical, radiological, or intraoperative evidence of cirrhosis were excluded.

After discharge, all patients underwent routine postoperative follow-up. Patients who had been managed by hepatologists before surgery continued hepatology follow-up after surgery. In addition, patients who developed worsening liver cirrhosis during follow-up were referred to hepatologists when necessary, and appropriate treatments were initiated. Nutritional therapy, including branched-chain amino acid (BCAA) supplementation, was administered according to the patient’s clinical condition.

The evaluated variables included liver cirrhosis status (assessed using the Child–Pugh classification), age, sex, pathological stage (pStage), surgical approach (laparotomy or laparoscopy), surgical procedure, degree of lymph node dissection, survival time, changes in serum total protein, albumin, prognostic nutritional index (PNI), and the liver volume from the preoperative period to 1 year postoperatively, and body composition at 1 year postoperatively, including the psoas muscle index (PMI), subcutaneous fat area, and visceral fat area at the level of the third lumbar vertebra (L3).

The psoas muscle area, subcutaneous fat area, visceral fat area, and liver volume were measured using a three-dimensional image analysis software program (SYNAPSE VINCENT; FUJIFILM, Tokyo, Japan) based on computed tomography images.

This study was approved by the Ethics Committee of the Kindai University Faculty of Medicine (approval No. 31–085). Informed consent was obtained from all patients through opt-out methods or written informed consent, in accordance with institutional guidelines.

### Statistical Analyses

Qualitative variables were compared using the chi-square test, and quantitative variables were analyzed using the Mann–Whitney U-test. In this study, we used 1:4 propensity score matching to control for measured confounders due to the retrospective nature of the study design. Propensity scores were estimated using logistic regression based on the pathological stage, surgical approach, and surgical procedure, which were considered potential confounders associated with postoperative complications. Propensity score matching was performed using the nearest-neighbor matching algorithm at a 1:4 ratio, with a caliper width of 0.2 of the standard deviation of the logit of the propensity score and without replacement. Common support was assessed by visually inspecting the propensity score distributions, which demonstrated an adequate overlap between the LC and non-LC groups. Baseline characteristics after propensity score matching were summarized using descriptive statistics, and covariate balance was assessed using standardized mean difference (SMDs). An SMD of less than 0.1 was considered indicative of an adequate covariate balance. As an additional multivariable analysis, Cox proportional hazards regression analysis was performed in the propensity score-matched cohort, adjusting for clinically relevant covariates, including age, sex, pathological stage, and lymph node dissection extent.

Survival curves were estimated using the Kaplan–Meier method, and group differences were assessed using the Cox proportional hazards model. The proportional hazards assumption was evaluated using the Schoenfeld residuals. Because a potential violation of the proportional hazards assumption was suggested, an exploratory piecewise Cox proportional hazards regression analysis was performed using a 1-year cutoff point based on the observed pattern of the Kaplan–Meier survival curves. Follow-up was restricted to 3 years because most survival events occurred within this period, and to further evaluate early postoperative mortality patterns. Hazard ratios (HRs) and 95% confidence intervals (CIs) were calculated for each time interval.

All analyses were performed using IBM SPSS Statistics (version 29; IBM Corporation, NY, USA) and EZR (version 1.70; Jichi Medical University, Tochigi, Japan). Statistical significance was set at *P* < 0.05.

## Results

The clinicopathological characteristics of the patients are presented in Table 1. Before propensity score matching (PSM), no statistically significant differences were observed between the two groups; however, imbalances were noted in sex distribution, surgical approach, operative time, and extent of lymph node dissection. After 1:4 PSM, the standardized differences for all covariates, except sex, were less than 0.1, indicating an adequate balance between the groups. Common support was assessed by visually inspecting the propensity score distributions, which demonstrated an adequate overlap between the LC and non-LC groups (Supplementary Figure SF1). Because the imbalance in sex persisted after matching, multivariable analyses, including the selected confounding factors, were additionally performed to evaluate prognosis (Supplementary Table ST1). In these analyses, the pathological stage and liver cirrhosis remained significant prognostic factors, whereas sex was not identified as an independent prognostic factor.

**Table 1:** Patient’s Backgrouds

	Before PSM				After PSM			
	LC group(*n*=25)	non-LC group(*n*=822)	Odds ratio [95%CI]	Std Dff.	LC group(*n*=25)	non-LC group(*n*=100)	Odds Ratio [95%CI]	Std Dff.
Age (years)	72.96±7.93	69.32±9.49	(※0.39 [-0.01-0.78] )	0.047	72.96±7.93	70.89±11.44	(※0.19 [-0.25-0.63] )	0.020
male/female	22/3	575/247	3.15 [0.93-10.62]	0.396	22/3	77/23	2.19 [0.60-7.98]	0.271
pStage								
Ⅰ	14	496	0.84 [0.38-1.87]	0.089	14	56	1.00 [0.414-2.42]	0
Ⅱ	6	168	1.23 [0.48-3.13]	0.088	6	24	1.00 [0.358-2.79]	0
Ⅲ	4	109	1.25 [0.42-3.70]	0.076	4	16	1.00 [0.303-3.31]	0
Ⅳ	1	49	0.66 [0.09-4.96]	0.081	1	4	1.00 [0.107-9.36]	0
Open / Laparocopic	10/15	383/439	1.31 [0.58-2.95]	0.132	10/15	36/64	0.84 [0.34-2.07]	0.083
Surgical approach								
DG	22	577	3.11 [0.92-10.50]	0.391	22	88	1.00 [0.26-3.85]	0.033
PG	1	90	0.339 [0.05- 2.54]	0.224	1	4	1.00 [0.11-9.36]	0
TG	2	155	0.37 [0.09-1.60]	0.279	2	8	1.00 [0.20-5.03]	0
Dissection (D1/D2)	20/5	521/301	0.43 [0.16-1.17]	0.346	20/5	80/20	1.00 [0.33-2.99]	0

PSM; 1:4 Propensity Score Matching 95%CI; 95%Confidence Interval ※Cohen’s d

**Fig. 1:**
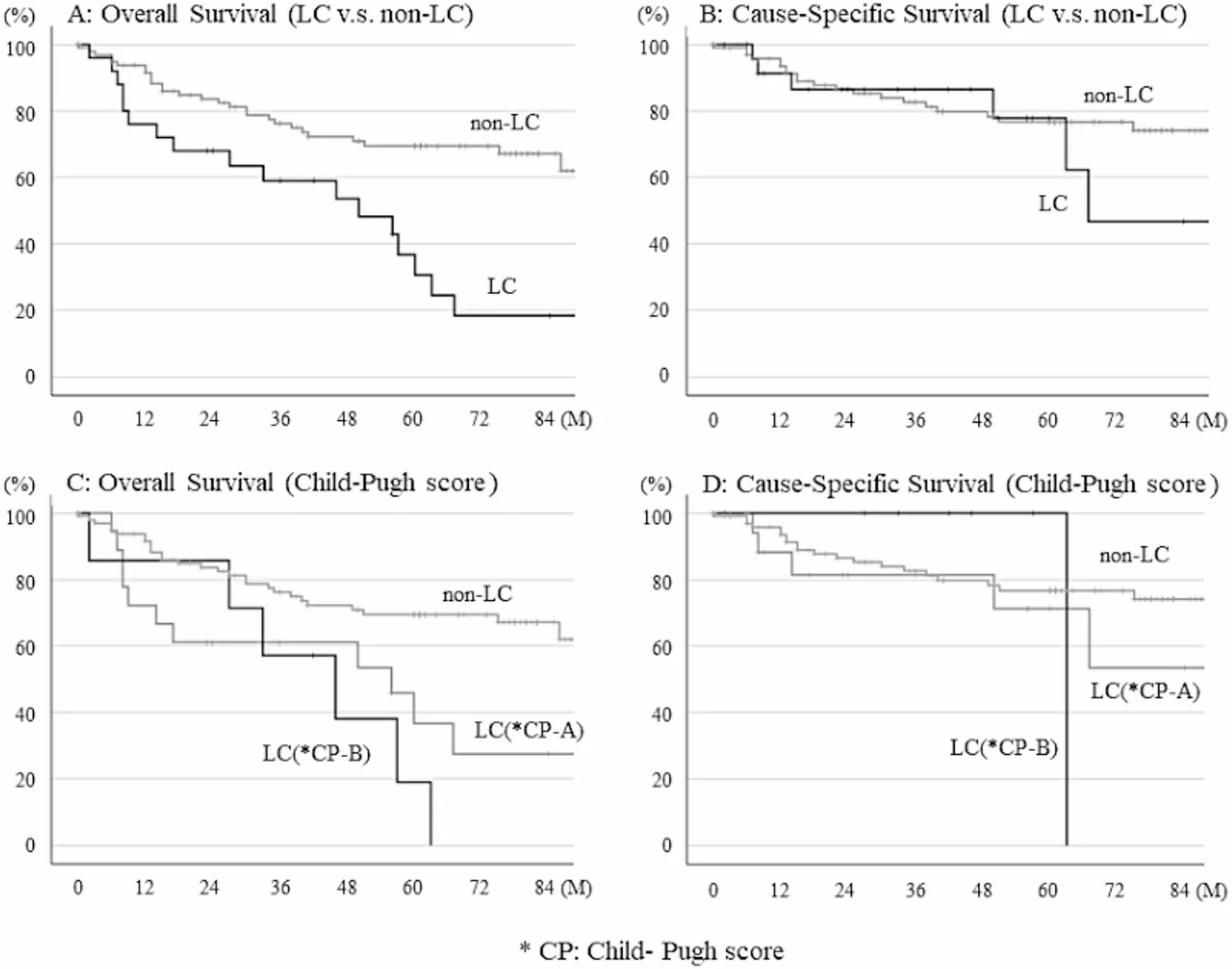
Kaplan–Meier Curves for Overall and Cause-Specific Survival

The prognostic outcomes assessed using a Kaplan–Meier survival analysis and Cox proportional hazards regression models are shown in Fig. 1; Table 2. The 5-year overall survival (OS) rate was 69.4% (3-year OS: 76.2%) in the non-LC group and 30.6% (3-year OS: 58.9%) in the LC group, with a significant difference between the two groups (Fig. 1A). As an additional analysis, a multivariable Cox proportional hazards regression analysis was performed in the propensity score-matched cohort. After adjusting for age, sex, pathological stage, and extent of lymph node dissection, liver cirrhosis remained independently associated with poorer overall survival (HR 2.853, 95% CI 1.755–4.639, *P* < 0.001) (Supplementary Table ST2). In contrast, the 5-year cancer-specific survival (CSS) rate was 76.7% (3-year CSS: 82.6%) in the non-LC group and 77.8% (3-year CSS: 86.5%) in the LC group, with no significant difference between the groups (Fig. 1B). When analyzed according to the Child–Pugh classification, the 5-year OS rates were 36.7% (3-year OS:61.1%) in Child–Pugh class A (CP-A) and 19.0% (3-year OS: 57.1%) in Child–Pugh class B (CP-B) (Fig. 1C). Compared with the non-LC group, both the CP-A and CP-B groups showed significantly poorer OS; however, no significant difference was observed between CP-A and CP-B. The 5-year CSS rates were 71.3% (3-year CSS: 81.4%) in CP-A and 100% (3-year CSS: 100%) in CP-B, and no significant differences were observed when compared with the non-LC group (Fig. 1D). Additionally, no significant differences in CSS were observed between CP-A and CP-B.

**Table 2:** Comparison of Overall and Cause-Specific Survival Using Cox proportional hazards regression model

	Cox proportional hazards regression model			
	OS		CSS	
	p value	HR[95％CI]	p value	HR[95％CI]
LC versus non-LC	<0.01	2.685 [1.47–4.91]	0.46	1.41 [0.56–3.52]
non-LC versus CP-A	0.02	2.387 [1.16–4.72]	0.34	1.62 [0.61–4.32]
non-LC versus CP-B	0.01	3.393 [1.392–8.27]	0.82	0.79 [0.11–5.89]
CP-A versus CP-B	0.37	1.601 [0.58–4.45]	0.8	0.75 [0.08–6.76]

OS; overall survival CSS; Cause-specific survival HR; Hazard ratio CP-A; Child- Pugh A CP-B; Child- Pugh

The Kaplan–Meier curves showed crossing between groups, and the proportional hazards assumption was evaluated using Schoenfeld residuals (Supplementary Figure SF2). Although most Schoenfeld residual tests were not statistically significant, the smoothed Schoenfeld residual plots deviated from a zero-slope horizontal line, suggesting a potential time-dependent effect. Log–minus–log plots also indicated a possible violation of the proportional hazards assumption approximately 1 year postoperatively (Supplementary Figure SF3). As a sensitivity analysis, a piecewise constant hazard model with a cutoff point at 1 year after surgery was applied, with the observation period restricted to 3 years postoperatively. The results showed significant differences in overall survival (OS) during the first postoperative year between the LC and non-LC groups and between the non-LC and CP-A groups. In contrast, no significant differences were observed in cancer-specific survival (CSS) among the groups (Table 3).

**Table 3:** Exploratory Piecewise Cox Regression Analysis of Overall and Cause-Specific Survival

	Time Interval	OS		CSS	
		HR[95%CI]	*p* value	HR[95%CI]	*p* value
LC versus non-LC	≤ 1year	2.99 [1.04–8.62]	0.04	1.34 [0.27–6.65]	0.72
	1-3yea	1.37 [0.45–4.21]	0.58	0.49 [0.06–3.89]	0.50
non-LC versus CP-A	≤ 1year	3.44 [1.13–10.53]	0.03	1.85 [0.37–9.19]	0.45
	1-3yea	1.04 [0.23–4.60]	0.96	0.62 [0.08-4.91]	0.65
non-LC versus CP-B	≤ 1year	1.81 [0.23–14.51]	0.57	0 [<0.01–>100]	0.97
	1-3yea	2.00 [0.45–8.85]	0.36	0.04 [0.00–>>100]	0.59
CP-A versus CP-B	≤ 1year	1.88 [0.22–16.07]	0.57	0 [ <0.01–>100]	0.96
	1-3yea	0.541 [0.08–3.86]	0.54	36.74 [0.00–>100]	0.69

In an exploratory analysis evaluating changes in serum total protein levels, albumin levels, and the prognostic nutritional index (PNI) from the preoperative period to one year after surgery, these parameters showed higher values in the cirrhosis group than in the non-cirrhosis group (Supplementary Figure SF4).

**Fig. 2:**
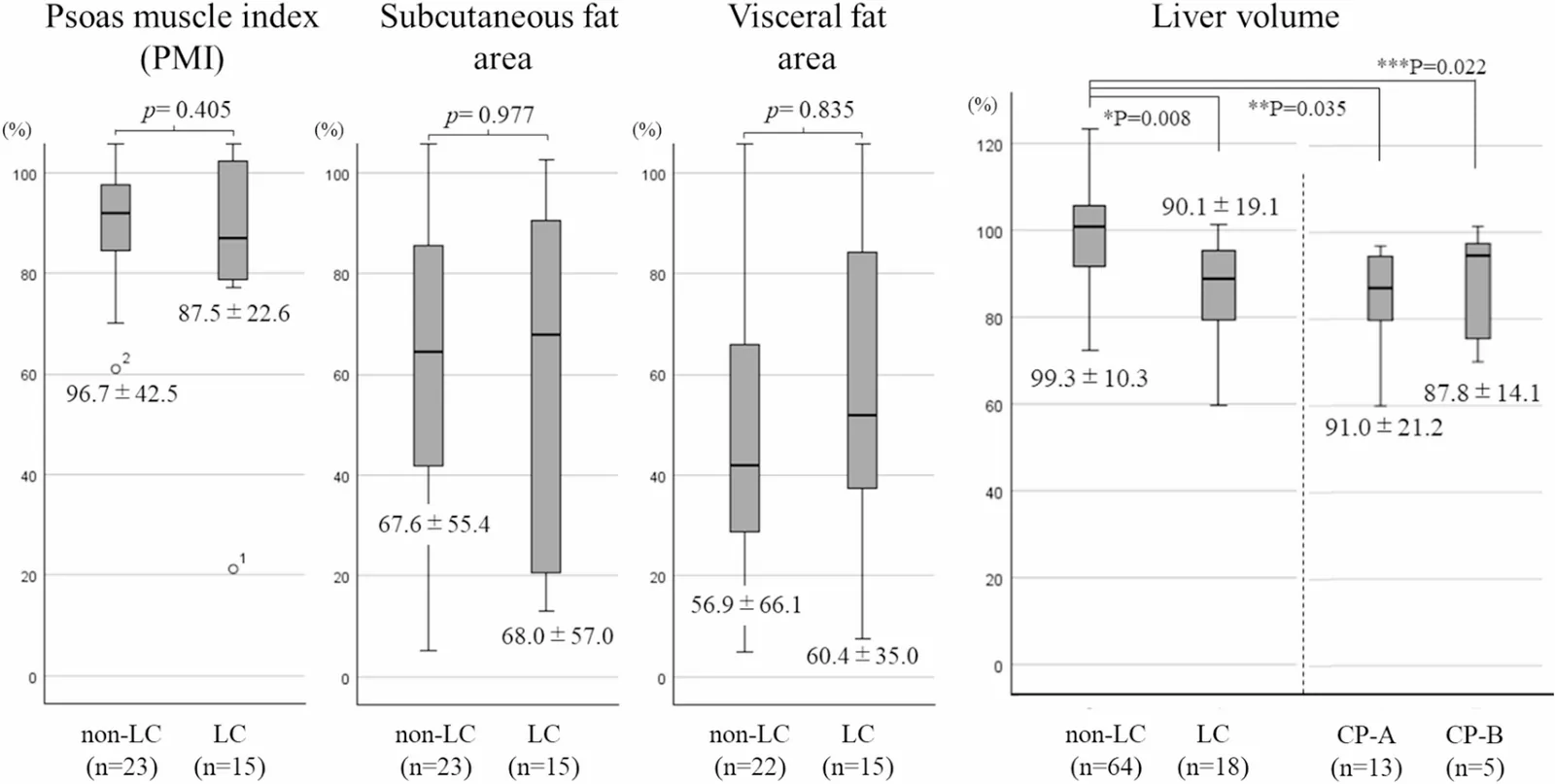
Pre- and postoperative changes in PMI, subcutaneous fat area, visceral fat area, and liver volume

In contrast, an analysis of the changes in the psoas muscle area (PMA), subcutaneous fat area, and visceral fat area from the preoperative period to one year after surgery demonstrated considerable postoperative reductions in all three body composition parameters in both the LC and non-LC groups. The mean reduction rates were approximately 5%, 35%, and 40%, respectively; however, the magnitude of these reductions did not differ significantly between the groups (Fig. 2).

In an exploratory analysis evaluating changes in liver volume between the preoperative period and 1 year postoperatively, the LC group showed a significantly higher proportion of patients with decreased liver volume than the non-LC group (Cohen’s d [95% CI], 0.723 [0.186, 1.255]). Furthermore, in the analyses stratified by Child–Pugh classification, a significant reduction in liver volume was observed not only in patients with CP-B (Cohen’s d [95% CI], 1.088 [0.156, 2.013]) but also in those with CP-A (Cohen’s d [95% CI], 0.653 [0.046, 1.256]) (Fig. 2).

Regarding the causes of death, the LC group had a higher proportion of deaths from non–gastric cancer–related causes (Table 4), with a significantly greater number of deaths attributable to liver cirrhosis and hepatocellular carcinoma. A time-dependent analysis of the causes of death showed that liver disease-related deaths occurred in three patients within 1 year after surgery (two due to liver cirrhosis and one due to hepatocellular carcinoma), in two patients between 1 and 3 years after surgery (both due to liver cirrhosis), and in four patients between 3 and 5 years after surgery (two due to liver cirrhosis and two due to hepatocellular carcinoma) (Table 5).

**Table 4:** Cause of death

Cause of death	non-LC: *n* = 100	LC: *n* = 25 (CP-A: *n* = 18/ CP-B: *n* = 7)	Odds ratio	95% CI	*p* value
primary cancer–related death	20 (20%)	6 (5 / 1) (24%)	1.26	0.44–3.63	*0.65*
Liver failure	0 (0%)	6 (3 / 3) (24%)	64.7	3.6–1158	<0.001
Hepatocellular carcinoma	0 (0%)	4 (4 / 0) (16%)	38.1	2.0–736	0.002
Cardiac disease	1 (1%)	0 (0%)	0.32	0.01–8.2	1
Respiratory disease	2 (2%)	1 (1 / 0) (4%)	2.04	0.18–23.4	0.53
Other malignancies	2 (2%)	0 (0%)	0.32	0.02–6.6	1
Other diseases	2 (2%)	2 (0 / 2) (8%)	4.24	0.56–32.1	0.18
Unknown	2 (2%)	0 (0%)	0.32	0.02–6.6	1

*Haldane–Anscombe correction

**Table 5:** Time-dependent analysis of causes of death

Cause of death	Within 1 year			1-3 y			3-5y			After 5y		
	CP-A (*n*=5)	CP-B (*n*=1)	Total (*n*=6)	CP-A (*n*=5)	CP-B (*n*=2)	Total (*n*=7)	CP-A (*n*=4)	CP-B (*n*=3)	Total (*n*=7)	CP-A (*n*=4)	CP-B (*n*=1)	Total (*n*=5)
Primary cancer–related death	2	0	2	1	0	1	1	0	1	1	1	2
Liver failure	2	0	2	1	1	2	0	2	2	0	0	0
Hepatocellular carcinoma	1	0	1	0	0	0	2	0	2	1	0	1
Respiratory disease	0	0	0	0	0	0	0	0	0	1	0	1
Other diseases	0	1	1	0	1	1	0	0	0	0	0	0

## Discussion

This retrospective analysis investigated the prognosis of patients with gastric cancer complicated by liver cirrhosis. To adjust for potential confounding factors, 1:4 propensity score matching (PSM) was performed. Although the imbalance in sex distribution between the two groups could not be completely eliminated, sex was not identified as an independent prognostic factor in the multivariate analysis. Therefore, the impact of the remaining imbalance in sex distribution on the study results is likely to be minimal. Nevertheless, the possibility of residual confounding cannot be completely excluded and should be acknowledged as a limitation of this study’s findings.

In Japan, where viral hepatitis and liver cirrhosis remain prevalent, advances in antiviral therapy and perioperative management have gradually improved the prognosis of patients with liver cirrhosis [11–14]. Consequently, gastric cancer with concomitant liver cirrhosis is increasingly encountered in routine clinical practice.

However, gastrectomy for patients with gastric cancer and concomitant liver cirrhosis may exacerbate portal hypertension due to surgical stress, including lymph node dissection, leading to postoperative ascites and paralytic ileus, and in severe cases, resulting in organ failure or sepsis and consequently a poor prognosis [15].

Against this background, several studies have evaluated surgical risk and prognosis using the severity classifications of hepatic dysfunction, including the Child–Pugh classification. Jang et al. reported inferior 5-year survival rates in patients with liver cirrhosis compared with those without cirrhosis [16]. Moreover, a cause-of-death analysis showed that gastric cancer recurrence predominated in patients with Child–Pugh class A, whereas liver cirrhosis–related causes, including hepatic failure and hepatocellular carcinoma, were more frequent in those with class B/C, indicating that aggressive surgery should not be pursued solely to achieve curative resection [16]. Similarly, Ikeda et al. demonstrated a poor prognosis in patients undergoing gastrectomy with concomitant liver cirrhosis, with significantly worse outcomes observed, particularly in Child–Pugh class B patients. They also highlighted the risk of metachronous hepatocellular carcinoma and emphasized that postoperative surveillance should focus not only on gastric cancer recurrence, but also on the early detection of hepatocellular carcinoma [17].

Collectively, these reports suggest that treatment strategies for gastric cancer in patients with liver cirrhosis should address not only surgical safety and curative intent but also comprehensive postoperative management, including the prevention and early intervention of hepatic dysfunction and hepatocellular carcinoma (HCC). However, most existing studies have primarily focused on passive or observational approaches to postoperative management, such as close monitoring, and few reports have proposed active interventional strategies. To establish more effective treatment strategies for patients with gastric cancer and liver cirrhosis, it is essential to obtain more detailed information regarding the extent and underlying causes of long-term postoperative hepatic functional impairment.

In the present study, we focused on the observation that many patients with Child–Pugh class A cirrhosis, who are generally expected to have favorable long-term survival after gastrectomy [18, 19], died relatively early from liver-related causes, including hepatic failure and hepatocellular carcinoma. Based on these findings, we hypothesized that gastrectomy may impose persistent hepatic stress, thereby accelerating the progression to hepatic failure or the development of hepatocellular carcinoma within a shorter period than expected. Accordingly, we sought to determine whether this cumulative hepatic burden was primarily attributable to surgical stress itself or to long-term postoperative consequences, particularly postoperative malnutrition, which may represent a potential target for preventive interventions.

Substantial evidence has accumulated regarding the impact of surgical stress on patients with cirrhosis. Limiting the extent of lymph node dissection, particularly by avoiding dissection around the hepatic hilum and common hepatic artery, has been widely recognized to improve surgical safety and reduce postoperative complications [20–23]. Recently, laparoscopic approaches and technical refinements in liver retraction have further reduced surgical invasiveness and postoperative hepatic injury [24–26]. Consequently, these strategies have been widely adopted and have contributed to lower postoperative complication rates in patients.

With these advances in perioperative management, gastrectomy is generally considered safe for patients with Child–Pugh class A cirrhosis. For example, Jang et al. reported a 5-year overall survival rate of 66% in patients with Child–Pugh class A cirrhosis compared to only 11% in those with Child–Pugh class B/C disease [16]. Similar outcomes have been reported by Isozaki et al. and Takeda et al. [27, 28].

Based on these previous studies, we initially expected that patients with Child–Pugh class A cirrhosis would achieve favorable long-term outcomes comparable to those previously reported. Contrary to our expectations, the long-term survival remained significantly worse, even in patients with Child–Pugh class A cirrhosis. Furthermore, the crossing of the Kaplan–Meier survival curves, Schoenfeld residuals, and visual inspection of the log-minus-log plots suggested a violation of the proportional hazards assumption approximately 1 year after surgery. Therefore, we performed a sensitivity analysis using a piecewise constant hazard model. The cutoff point was set at 1 year postoperatively based on previous studies demonstrating that food intake and body weight undergo substantial changes during the first postoperative year and subsequently stabilize [11–13], together with the findings from log-minus-log plots. In addition, to evaluate the impact of gastrectomy itself while minimizing the influence of long-term liver-related events attributable to the natural course of cirrhosis, the observation period was restricted to the first three postoperative years.

The piecewise constant hazard analysis demonstrated a temporal change in the hazard ratio approximately 1 year after surgery. Moreover, liver cirrhosis- or liver disease-related deaths occurred within 3 years after surgery, even among patients with Child–Pugh class A or B cirrhosis. These findings suggest that even patients with an apparently well-preserved liver function may experience progressive deterioration of hepatic function or hepatic reserve relatively early after gastrectomy, which may subsequently affect their long-term survival.

Interestingly, despite the early separation of the survival curves, all patients recovered sufficiently to be discharged after surgery, and routine follow-up revealed no persistent clinical signs of postoperative deterioration. Therefore, postoperative metabolic and nutritional changes may represent one of several possible contributing factors; however, the present observational study cannot establish a causal relationship, and the contribution of other mechanisms cannot be excluded.

One possible explanation for this phenomenon is the postoperative metabolic and nutritional changes resulting from impaired digestive and absorptive functions after gastrectomy. Nutritional impairment remains one of the major long-term complications after gastrectomy, whereas in patients with cirrhosis, protein-energy malnutrition and energy deficiency are well-established risk factors for hepatic deterioration, hepatic decompensation, increased complication rates, and a poor survival [29–31]. Therefore, during the first postoperative year, when nutritional impairment is most pronounced, deterioration in digestive and absorptive function may impose an additional metabolic burden on the vulnerable physiological state of patients with cirrhosis, which may be associated with the deterioration of the hepatic reserve or progression of cirrhosis. This hypothesis may partly explain the early divergence of the survival curves observed in the present cohort.

Therefore, we performed exploratory analyses of changes in serum total protein, albumin, and prognostic nutritional index (PNI) levels from the preoperative period to 1 year postoperatively. In addition, exploratory analyses of the psoas muscle index (PMI), subcutaneous fat area, visceral fat area at the level of the third lumbar vertebra (L3), and liver volume were conducted using computed tomography (CT) images. Given the limited sample size, particularly in the Child–Pugh subgroup analysis, these exploratory findings should be interpreted with caution.

In the present study, contrary to our expectations, the serum total protein levels, albumin levels, and PNI were better preserved in the cirrhosis group during the first postoperative year (Supplementary Figure SF4). However, the serum albumin levels in patients with cirrhosis are influenced by various factors, including hepatic synthetic function, systemic inflammation, fluid retention, and ascites [30, 32–34]. Accordingly, recent guidelines and reviews have highlighted the limitations of albumin-based nutritional assessment in patients with cirrhosis and emphasized the importance of body composition analysis [30, 32–34]. Furthermore, the albumin and PNI levels may be affected by postoperative albumin supplementation, fluid shifts associated with diuretic therapy, dehydration-induced hemoconcentration, and cirrhosis-associated hypergammaglobulinemia [35]. Therefore, the apparently favorable postoperative changes in these serological markers may not necessarily reflect the true nutritional and metabolic status of patients with cirrhosis. In contrast, CT-based body composition parameters, including the psoas muscle, subcutaneous fat, and visceral fat areas, may provide a more direct assessment of the nutritional reserves.

The rates of decrease in the psoas muscle index (PMI), subcutaneous fat area, and visceral fat area were approximately 5%, 35%, and 40%, respectively, which is consistent with previous reports [36–39]. Although no statistically significant differences were observed between the patients with and without liver cirrhosis, substantial reductions in the skeletal muscle and adipose tissue were observed in both groups. Such marked changes in body composition are unlikely to occur over a one-year period in the natural course of non-surgical patients and may reflect the profound nutritional and metabolic consequences of gastrectomy.

Previous studies by Carey et al. and Montano-Loza et al. demonstrated that sarcopenia is an independent predictor of a poor prognosis [40, 41]. Furthermore, Hanai et al. reported that an annual skeletal muscle loss rate exceeding 3.1% is associated with adverse outcomes [42]. In the present study, the observed reduction in PMI exceeded this threshold, suggesting clinically relevant muscle loss following gastrectomy. Taken together, these findings demonstrate substantial postoperative changes in body composition after gastrectomy; however, their contribution to hepatic atrophy and long-term outcomes, particularly in patients with cirrhosis remains unclear. Although nutritional deterioration has been proposed as a potential mechanism underlying poor postoperative outcomes, our findings did not establish a causal relationship between the postoperative nutritional changes and liver-related mortality. Further prospective studies are warranted to clarify whether nutritional or other perioperative interventions improve the long-term outcomes of patients with cirrhosis.

To our knowledge, no previous study has directly examined whether a reduction in liver volume reflects the natural progression of cirrhosis. However, the natural history of Child–Pugh A cirrhosis is generally considered to be relatively favorable. In a Japanese multicenter study, the 5-year survival rate was reported to be 61% in Child–Pugh A patients, compared with 42% in Child–Pugh B patients and 25% in Child–Pugh C patients [43]. Similarly, a nationwide analysis including 57,769 patients demonstrated a favorable 3-year survival rate of 75.3% among Child–Pugh A patients [44]. Furthermore, D’Amico et al. reported a median survival exceeding 12 years in patients with compensated cirrhosis [18], whereas Christensen et al. reported a median survival of 6.4 years in patients with a Child–Turcotte score of 5, which roughly corresponds to Child–Pugh A cirrhosis [19]. Collectively, these findings indicate that the prognosis of nonsurgical Child–Pugh A cirrhotic patients is generally favorable.

In contrast, the survival outcomes of Child–Pugh A patients in our gastrectomy cohort were inferior to those reported for the natural history of cirrhosis. These findings suggest that the postoperative changes associated with gastrectomy, including liver volume reduction and metabolic or nutritional changes, may be one of several factors associated with the poorer long-term outcomes observed in our cohort. However, the present observational study was unable to directly demonstrate a causal relationship.

Additionally, our results differed from those of previous reports, as a relatively high rate of postoperative liver-related mortality was observed even among patients with Child–Pugh class A, potentially reflecting the limitations of the Child–Pugh classification in assessing hepatic functional reserve. Although the Child–Pugh classification remains widely used and clinically useful, it is well recognized that it includes subjective components and has limited discriminatory ability [45–47]. Indeed, Zhao et al. demonstrated in a comparative study with the albumin–bilirubin (ALBI) grade that substantial prognostic heterogeneity exists even within the Child–Pugh class A population, highlighting the need for re-stratification using more objective indices [47].

Taken together, these findings suggest that some patients have a subclinical impairment of the hepatic functional reserve that cannot be adequately captured by the Child–Pugh classification alone. In such patients, the physiological stress associated with surgery may exceed the hepatic compensatory capacity, potentially contributing to impaired long-term outcomes. Therefore, careful patient selection and rigorous preoperative assessment are warranted when determining the indications for gastrectomy in patients with liver cirrhosis.

Furthermore, the combined use of additional liver function assessment tools alongside the Child–Pugh classification may enable a more sensitive evaluation of hepatic functional reserve, which could contribute to the selection of more appropriate surgical strategies for patients with HCC.

### Limitations

This study has several limitations. First, the relatively small number of patients with liver cirrhosis (*n* = 25) may have reduced the statistical power, increased the risk of type II error, and resulted in unstable effect estimates with wide confidence intervals. The limited number of events also restricted the extent of multivariable adjustment and raised the possibility of residual confounding. Second, the retrospective, single-center design may have introduced a selection bias. Although propensity score matching achieved adequate balance for most covariates, residual confounding due to unmeasured variables cannot be completely excluded, and residual imbalance in sex distribution remained after matching. Furthermore, the observational design of this study does not allow for causal inferences regarding the relationships among gastrectomy, postoperative nutritional deterioration, liver atrophy, and liver disease-related mortality. Therefore, the observed associations should be interpreted with caution and should not be considered evidence of a causal pathway. However, sensitivity analyses, including the piecewise constant hazard model, yielded consistent results. Therefore, while the present findings provide clinically relevant insights into the long-term outcomes after gastrectomy in patients with liver cirrhosis, they should be interpreted with caution and require validation in larger multicenter prospective studies.

## Conclusion

In conclusion, patients with liver cirrhosis had a significantly worse overall survival after gastrectomy for gastric cancer, primarily because of liver disease-related mortality, whereas the cancer-specific survival was comparable to that of patients without cirrhosis. Patients with cirrhosis also exhibited a greater postoperative reduction in liver volume; however, the mechanisms underlying this finding and its clinical significance remain unclear. Further prospective studies are warranted to improve risk stratification and determine whether perioperative interventions, including nutritional management, can improve the postoperative outcomes.
